# Female sterility associated with increased clonal propagation suggests a unique combination of androdioecy and asexual reproduction in populations of *Cardamine amara* (Brassicaceae)

**DOI:** 10.1093/aob/mcv006

**Published:** 2015-03-16

**Authors:** Andrew Tedder, Matthias Helling, John R. Pannell, Rie Shimizu-Inatsugi, Tetsuhiro Kawagoe, Julia van Campen, Jun Sese, Kentaro K. Shimizu

**Affiliations:** ^1^Institute of Evolutionary Biology and Environmental Studies and Institute of Plant Biology, University of Zurich, Winterthurerstrasse 190, CH-8057, Switzerland, ^2^Department of Ecology and Evolution, University of Lausanne, Lausanne CH-1015, Switzerland, ^3^Center for Ecological Research (CER), Kyoto University, 2-509-3, Hirano, Otsu, Shiga 520-2113, Japan and ^4^Computational Biology Research Center (CBRC), National Institute of Advanced Industrial Science and Technology (AIST) Koto-ku, Tokyo, 135-0064, Japan

**Keywords:** *Cardamine amara*, Brassicaceae, *Arabidopsis thaliana*, female sterility, androdioecy, clonal propagation, reduced male fertility, asexual reproduction

## Abstract

**Background and Aims** The coexistence of hermaphrodites and female-sterile individuals, or androdioecy, has been documented in only a handful of plants and animals. This study reports its existence in the plant species *Cardamine amara* (Brassicaceae), in which female-sterile individuals have shorter pistils than seed-producing hermaphrodites.

**Methods** Morphological analysis, *in situ* manual pollination, microsatellite genotyping and differential gene expression analysis using *Arabidopsis* microarrays were used to delimit variation between female-sterile individuals and hermaphrodites.

**Key Results** Female sterility in *C. amara* appears to be caused by disrupted ovule development. It was associated with a 2.4- to 2.9-fold increase in clonal propagation. This made the pollen number of female-sterile genets more than double that of hermaphrodite genets, which fulfils a condition of co-existence predicted by simple androdioecy theories. When female-sterile individuals were observed in wild androdioecious populations, their ramet frequencies ranged from 5 to 54 %; however, their genet frequencies ranged from 11 to 29 %, which is consistent with the theoretically predicted upper limit of 50 %.

**Conclusions** The results suggest that a combination of sexual reproduction and increased asexual proliferation by female-sterile individuals probably explains the invasion and maintenance of female sterility in otherwise hermaphroditic populations. To our knowledge, this is the first report of the coexistence of female sterility and hermaphrodites in the Brassicaceae.

## INTRODUCTION

The occurrence of female-sterile individuals (functional males) in hermaphroditic populations is extremely rare; in sexually reproducing populations this dimorphism is known as androdioecy (Darwin, 1877; Charlesworth, 1984; Pannell, 2002). Theoretical models of androdioecy explain its rarity by predicting that males must sire at least twice as many successful progeny as hermaphrodites (Lloyd, 1975*a*; Charlesworth and Charlesworth, 1978, 1981; Charlesworth, 1984). Despite these rather stringent conditions, several examples of androdioecious plants have been well documented [e.g. *Datisca glomerata* (Liston *et al.*, 1990; Fritsch and Rieseberg, 1992; Weller and Sakai, 1999; Wolf *et al.*, 2001); *Sagittaria lancifolia* (Muenchow, 1998) and *Mercurialis annua* (Pannell, 1997)]; interestingly, most of these examples appear to have evolved from dioecy through the spread of pollen-producing females rather than from a hermaphroditic ancestor through the spread of female-sterility mutations (reviewed in Pannell, 2002).

Although the empirical evidence suggests that androdioecy is more likely to evolve from dioecy through the evolution of a male function in females, female sterility does appear to have invaded hermaphroditic populations in several species of plant. The olive family, Oleaceae, provides a particularly interesting case (Wallander, 2001), in which androdioecy appears to have evolved from hermaphroditism a number of times through the spread of female-sterile mutations. In the androdioecious species *Phillyrea angustifolia*, variation in the frequency of males with hermaphrodites (Aronne and Wilcock, 1992; Lepart and Dommée, 1992; Traveset, 1994) was hypothesized by Pannell and Ojeda (2000) to be partly the result of trade-offs in resource allocation between seed and pollen production. More recently, Samitou-Laprade *et al*. (2010) showed that the unusual linkage between a diallelic self-incompatibility locus and a locus determining sex immediately explains the maintenance of males with hermaphrodites at the high frequencies observed in wild populations (see also Vassiliadis *et al*., 2000, 2002: Pannell and Korbecka, 2010).

Trade-offs between sexual and clonal reproduction could also account for the maintenance of female-sterile individuals with hermaphrodites. For instance, female sterility has also been described in the subarctic species *Saxifraga cernua* and *S. foliolosa* (Molau, 1992; Molau and Prentice, 1992), in which it seems that increased clonal propagation by plants that do not produce seeds could explain the maintenance of female sterility (Molau, 1992). However, it is unclear whether males play an important role in siring seeds in these species, as meiosis shows irregularities and reproduction is predominantly asexual (Molau and Prentice, 1992; and see Muenchow, 1998). A similar trade-off between seed production by hermaphrodites and increased clonal growth by female-sterile individuals was invoked to explain the maintenance of males in Spanish populations of *Ulmus minor* (López-Almansa *et al*., 2002). In this case, the polymorphism might be maintained by a balance between, on the one hand, sexual reproduction and the colonization by seedlings of open habitat after flood disturbance and, on the other, clonal propagation in the absence of disturbance.

In this paper we document and interpret a new case of female sterility in an otherwise hermaphroditic plant, in which trade-offs in resource allocation between sexual and asexual reproduction appear to be important. A survey of Swiss populations of the subaquatic clonal mustard *Cardamine amara* revealed the frequent occurrence of female-sterile individuals with reduced pistil length, due to apparently impaired pistil development and a lack of seed production. Here we examine various aspects of the morphology and reproductive biology of *C. amara* populations. Specifically, we: (1) characterize variation in floral morphology between the phenotypic sexes with a view to clarifying whether reduced pistil length is an adequate indicator of female sterility; (2) delimit the frequency of female sterility and the extent to which female-sterile individuals occasionally produce seeds (leakiness or gender plasticity); (3) clarify the pollen fertilities of hermaphrodites and female-sterile individuals at the ramet and genet levels, along with the capacity of hermaphrodites to self-fertilize; and (4) examine the possibility that allocation trade-offs between seed production and relative clonal propagation are sufficient to maintain female-sterile individuals in androdioecious populations. Given the relatively close phylogenetic relationship of *C. amara* to the model plant species *Arabidopsis thaliana*, we applied existing *Arabidopsis* microarrays to study genome-wide expression pattern in immature pistils. Female sterility is not known in any species closely related to a model of molecular genetics, and very little is known about its molecular and developmental genetics. Finally, we discuss whether this female sterility in *C. amara* might be interpreted as a new case of androdioecy.

## MATERIALS AND METHODS

### Study species and populations

The *Cardamine amara* (Brassicaceae) species complex has a broad, Eurasian distribution and comprises four diploid subspecies (subsp. *Amara*, subsp. *opicii*, subsp. *balcanica* and subsp. *pyrenaea*) and two tetraploid subspecies (subsp. *austriaca* and subsp. *olotensis*) (Marhold *et al*., 2002; Lihová *et al*., 2006*a*, *b*). *Cardamine amara* subsp. *amara* (large bitter-cress) is a large (10–60 cm) perennial herb that is widespread across most of central Europe, extending into parts of Asia (Marhold, 1998; Lihová *et al*., 2000). It has an ascending stem and a creeping stolon that produces rhizomes (up to 50 cm in length) that typically root at the nodes and allow efficient propagation by vegetative means. The subspecies is primarily restricted to wet areas, including river banks, fens and wet woodland, and as such is likely affected by periodic flooding (Koch *et al*., 2003). At low altitudes in Switzerland (<1000 m), *C. amara* is reported to be diploid (2*n* = 16), but polyploid populations of subsp. *austriaca* (2*n* = 32) are known at higher altitudes (>1000 m) (Neuffer and Jahncke, 1997; Urbanska *et al*., 1997; Marhold, 1998; Mandakova *et al*., 2013; Zozomová-Lihova *et al*., 2014). Lowland and higher-altitude populations flower between April and June and between May and July, respectively. Individual inflorescences consist of ∼10–130 flowers, with a single pistil, four obovate petals (typically white) and four sepals. Fruit and seed set are highly variable (Rich and Dalby, 1991). As is typical within the Brassicaceae, anthers exhibit the tetradynamous condition (four long medial and two short lateral anthers in each flower; Endress, 1992; Zomlefer, 1994).

We studied two diploid Swiss populations of *C. amara* located along riverbanks at Küssnachter Tobel (KTP) and Wehrenbach (WP), with additional estimates of female-sterile and hermaphrodite frequencies taken in two further populations: Goldach (GP) and Ticino (TP) (Table 1). The habitats at each of the four populations varied in the degree of human-mediated disturbance. The rivers at KTP, WP and GP have all undergone the addition of man-made structures, such as reinforced banks and/or waterfalls. Plants in the TP population grew in a small canal that ends in the Ticino River. For this reason, all four populations might best be characterized as semi-natural.

**Table 1.** Sample populations and estimation of frequencies of female-sterile individuals. For the two populations Küssnachter Tobel and Wehrenbach, subsets of plants were chosen for in-depth analysis of floral morphology

Population	Latitude (N)	Longitude (E)	*n*	Hermaphrodites	Female-sterile	Frequency of female sterility (%)	Date sampled
Küssnachter Tobel	47·31	8·63	248	234	14	5·65	May 2009
Küssnachter Tobel focal*	47·31	8·63	77	56	21	27·0	May 2009 and May 2010
Wehrenbach	47·36	8·56	114	52	62	54·4	April 2011
Wehrenbach focal*	47·36	8·56	86	41	45	52·3	April 2010 to April 2011
Goldach	47·41	9·48	187	177	10	5·35	May 2010
Ticino	46·51	8·67	51	51	0	0·00	May 2011

*These populations were sampled non-randomly to ensure adequate female-sterile sampling.

### Sample selection

**Fig. 1.**
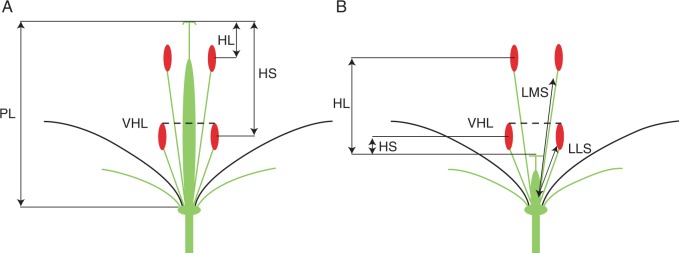
Diagram illustrating the floral traits measured in this study and the method by which phenotypic sex was determined. Flowers are viewed as a dissection. PL, pistil length; HL, herkogamy of medial stamen (stigma–mid-anther separation of the four medial stamens); HS, herkogamy of lateral stamen (stigma–mid-anther separation of the two short stamens); LMS, length of medial stamen; LLS, length of lateral stamen; VHL, virtual horizontal line used to determine phenotypic sex. If the stigma was above this line (A) the flower was assumed to be hermaphrodite. If the stigma fell below this line (B) it was assumed to be female-sterile.

Ramets from both of the focal populations (KTP and WP) were sampled at 2-m intervals to reduce the likelihood of sampling multiple ramets within any single genet that might be connected physically; this distance was increased if patch size was continuous for >2 m. Only ramets in flower at the time of sampling were selected. Sampled flowers with a stigma below or above a virtual horizontal line (VHL; Fig. 1) formed by the two lateral anthers were classified as ‘female-sterile’ (FS) and ‘hermaphrodite’ (H), respectively. Subsets of focal ramets, used for detailed analysis of floral morphology, were marked with a stake to ensure they could be relocated for later sampling (Table 1). In the WP population, in which the morph ratio was approximately 1 : 1, 86 focal individuals were sampled randomly (April 2011). In the KTP population, where there was a minority of FS individuals, we sampled 77 (May 2009 and May 2010) ramets non-randomly to ensure relatively equal representation of the two morphs.

### Morphological measurements

In the natural environment, we removed and measured five fresh mature flowers for each sampled ramet (i.e. flowers with completely open anthers), avoiding the first three flowers on each stem as a precaution; these flowers are often infertile in *Arabidopsis thaliana* (Weigel and Glazebrook, 2002). Flowers were either measured directly or fixed in 70 % ethanol for later measurement in the laboratory (no difference in measurements taken after fixation were observed).

We used digital callipers to measure the following morphological characteristics for each sampled individual in the KTP and WP populations: stamen length (distance between floral base and the mid-anther of the four medial stamens (medial stamen length) and the two lateral stamens (lateral stamen length); pistil length (distance between floral base and stigma); and stigma–anther separation (‘herkogamy’) between the stigma and the medial (HL) and lateral stamen (HS).

Morphological measurements were also used to assess floral development under controlled laboratory conditions for 4 successive days after flower opening. Plants were maintained under the following growth conditions: 14 h light (18 °C) and 10 h dark (16 °C) with 60 % humidity in a Sanyo (model MLR-351H) growth chamber. We observed five flowers per ramet for 20 flowering ramets (10 FS and 10 H). As before, the first three flowers on each stem were avoided as a precaution (Weigel and Glazebrook, 2002). 

### Male and female fertility in the natural environment

To assess the fitness of both FS and H individuals, we selected a subset of 15 H and 15 FS ramets from WP and recorded the following variables: total number of flowers per ramet; average number of pollen grains per flower (five flowers per ramet); and total number of seeds per ramet. Seed number was calculated using ripe siliques from each flower, with the exception of those used for pollen counting.

Pollen counts for all individuals were conducted using six anthers per individual, which were dissected from immature flowers, transferred into 20 ml of a solution of 50 % ethanol and 5 % Tween-80 (Fisher Scientific, Fair Lawn, NJ, USA), and incubated for 24 h at room temperature. The solution was then sonicated for 15 min and briefly vortexed to tear the anthers and release their pollen (Fulkerson *et al*., 2012). Ten millilitres of the suspension was then transferred to a Neubauer counting chamber (0·1 mm × 0·0025 mm^2^; Marienfeld Laboratory Glassware, Germany), and pollen grains were counted twice on different grids under a microscope with ×200 magnification. 

### Assessment of female sterility: pollination

Cross-pollinations were conducted for subsets of both populations *in natura* (*in situ*) (Shimizu *et al*., 2011), using 11 FS and 16 H individuals at KTP and 15 FS and 15 H individuals at WP. For each paternal individual (pollen donor), two pollen recipients (maternal individuals) were chosen. Pollen donors were crossed with recipients of each pistil morph to form a crossing set, with approximately five separate donors. With four exceptions (individuals FS1, FS2, FS7 and FS11), each crossing set was independent. Pollinations were carried out from April 2009 to May 2010 on an *ad hoc* basis, depending on flower availability. On selection of individuals, all open flowers were removed and the inflorescences were bagged. Cross-pollinations were performed on flowers emasculated prior to the anthers dehiscing. After pollination, bags were replaced to prevent external pollination. The following manual pollinations were performed (in the direction of female × male): H × H (self), H × H (outcross), H × FS, FS × H, FS × FS (self) and FS × FS (outcross). When an FS individual was the female parent, its short pistils were pollinated. In addition to the manual pollinations, we also tested seed set by autonomous self-pollination using control bag treatment. Fruits were collected and seed number per fruit was recorded throughout July 2009 and July 2010. We calculated the average number of seeds as well as the proportion of siliques with at least one seed, as many siliques had no seeds.

As pollen viability and compatibility have the potential to influence tests of female sterility, pollen-tube growth from donor individuals was tested for a subset of individuals using epiflorescence microscopy. Plants were grown under the following conditions: 14 h light (18 °C) and 10 h dark (16 °C) with 60 % humidity in a Sanyo growth chamber. Fresh flowers were collected on the day of anthesis and used for the pollination assay. Hand-pollinated pistils were cut at the peduncle 12 h after pollination. After fixation for 2 h in ethanol : acetic acid (3 : 1), the pistils were softened in 1 m NaOH at 60 °C for 1·5 h and stained with 0·01 % (w/v) decolorized aniline blue (Martin, 1959) for 2·5 h in 2 % (w/v) K_3_PO_4_. Pistils were gently squashed onto a microscope slide by placing the cover glass over the pistils, as described by Tsuchimatsu *et al.* (2012). Samples were examined under a fluorescence microscope (Leica DM6000) with an excitation filter of 395 nm and an emission filter of 420 nm (Dumas and Knox, 1983).

### Assessment of female sterility: ovule number

Ovule number and ovule size were determined using light microscopy. Pistils were fixed for a minimum of 2 h in acetic acid : ethanol (9 : 1). After fixation, pistils were washed in 90 % ethanol for 5 min, followed by a further 5 min in 70 % ethanol, and in Hoyer’s solution (Liu and Meinke, 1998) as described by Shimizu *et al.* (2008). The pistils were mounted on a glass slide and microscopic analysis was performed using a Leica DM6000 microscope with a magnification of ×200. All ovules in five siliques per individual were counted and photographs were taken. Ovule area was determined using the program ImageJ (W. S. Rasband, ImageJ, National Institutes of Health, Bethesda, MD, USA; http://imagej.nih.gov/ij/, 1997-2011). To delimit ovule phenotype for FS and H, one fruit per individual, for 10 individuals per morph, were analysed under the microscope with ×400 magnification, and photographs were taken.

### Assessment of the mating system and clonal propagation

We used microsatellite markers to assess the mating system and the extent of clonal propagation. Leaf tissue was collected from all focal ramets at both WP (*n* = 47) and KTP (*n* = 71) between April 2009 and April 2011, and total genomic DNA was extracted from 100 mg of desiccated leaf tissue using DNeasy kits (Qiagen Inc.). Eight microsatellite loci, comprising six developed for the model species *Arabidopsis lyrata* [AthZFPG, ATTS0392, F20D22, ICE12, ICE14 and LYR104 (Clauss *et al*., 2002)] and DnA207 and DnA218 (Skrede *et al*., 2009), were screened for variation. Products were amplified by singleplex polymerase chain reaction (PCR), using the default reagent concentrations recommended by the manufacturer (Qiagen Inc.). Thermocycling was performed on PTC-200 (MJ Research) machines using the following programme: initial denaturation at 95 °C for 15 min followed by 34 cycles of 94 °C for 30 s, 55 °C for 90 s (ramp to 72 °C at 0·7 °C/s) and a final 72 °C extension for 10 min. Multiplex products (1 : 140 dilutions) were genotyped using an ABI 3730 sequencer. Genotypes were analysed using GENEMAPPER 4.0 (Applied Biosystems) and corrected manually. Using only unique multilocus genotypes (so as to prevent any potential bias created by the inclusion of duplicate genotypes), the inbreeding coefficient (*F*_IS_), observed (*H*_O_) and expected (*H*_E_) heterozygosity and deviation from Hardy–Weinberg equilibrium were calculated using GENEPOP v4.1 (Rousset, 1997). The effective selfing rate, accounting for any effects of inbreeding depression (*S*), was estimated from *F*_IS_ using the classical formula: *S* = (2 × *F*_IS_)/(1 + *F*_IS_). Estimation of *S* in this way assumes the population to be at equilibrium while providing an average selfing rate over several generations. However, if bi-parental inbreeding is occurring, selfing rates derived using this formula may be overestimated; conversely, if selection operates against homozygous genotypes, the selfing rate may be underestimated.

To assess whether samples with identical genotypes were the result of asexual reproduction, we estimated allelic frequencies using the method described by Parks and Werth (1993), in which subsampling is used to remove upward bias towards rare alleles that may distinguish genets. Unique genotype probabilities (*p*_gen_) were then calculated, and the probability that the same genotype was the result of distinct reproductive events (*p*_sex_) was estimated (Parks and Werth, 1993; Arnaud-Haond *et al*., 2005). This method relies on several assumptions, namely: (1) unique genotypes originate from a zygote rather than by mutation of an existing genotype; (2) mating is random [this may be violated in clonal populations (Cook, 1983)]; and (3) genotypes at separate loci are independent.

Clonal diversity was estimated in two ways, first by calculating the mean of identical genotypes (clones) for each morph and population, and second by calculating clonal diversity (*R*) for each morph within each population (Arnaud-Haond *et al.*, 2005), using the formula *R* = *G* – 1/*N* – 1, where *G* is the number of unique genotypes and *N* is the sample size. Finally, from observed ramet frequencies and unique multilocus genotypes, we calculated per genet sexual phenotype frequencies (genet frequencies) to assess the contribution of each sexual phenotype to the population.

### Comparison of gene expression between morphs

Taking advantage of the fact that *C. amara* is a close relative of *A. thaliana* (Mitchell-Olds, 2001; Shimizu and Purugganan, 2005; Shimizu and Purugganan, 2006; Morinaga *et al*., 2008; Shimizu *et al*., 2011), we examined differential gene expression between the two morphs using the *Arabidopsis* Gene Expression Microarray, 4x44k (Agilent, Santa Clara, USA). Progenies from a single mother plant originating from KTP were grown under the following conditions: 14 h light (18 °C) and 10 h dark (16 °C) with 60 % humidity in a Sanyo growth chamber; two H and three FS plants flowered despite a low germination rate. One of the H individuals was subjected to two technical replicates to obtain three H samples. We harvested pistils from flower buds with a length of 3·2–3·6 mm, at which the difference in pistil length between H and FS plants was visibly discernible. From each individual, ten pre-mature pistils were removed and directly frozen in liquid nitrogen. Total RNA was extracted using an RNeasy Plant Mini Kit (Qiagen, Hilden, Germany) according to the manufacturer’s protocol. The integrity of RNA was evaluated using a Bioanalyser 2100 (Agilent, Santa Clara, USA); RNA integrity in each sample was sufficient for gene expression analysis (RNA integrity number ≥9). Total RNA was labelled with a Quick Amp Labeling Kit (Agilent) and hybridized to the *Arabidopsis* (V4) Gene Expression Microarray, 4x44 (Agilent). Feature extraction and image analysis software (Agilent Technologies) was used to locate and delineate each spot in the array.

Data analysis was performed using Bioconductor software (v. 2.12.2) implemented in R v. 2.13.1 (http://www.r-project.org/). Normalization was performed according to the manual (Agilent Technologies) by moving the third quantile value of each sample to 10. As the microarray was heterologous, probes that had not hybridized sufficiently to the microarray chip were filtered (flag value <1). Prediction analysis for microarrays (PAM) was performed with the remaining 11 900 genes and ranked according to their differential expression using the Bioconductor package PAMR v. 1.54 (Tibshirani *et al*., 2002). Genes expressed differentially between H and FS were determined using a log_2_ fold change of ≥3·0. Gene ontology (GO) enrichment was tested with Fisher’s exact test, using the Bioconductor package topGO (Alexa *et al*., 2006) v. 2.2.0. The reference set consisted of all genes mapped by the probe set of the microarray used. The ‘elim’ algorithm of topGO was used to eliminate the hierarchical dependency of the GO terms, and FDR correction for multiple testing was applied using the Benjamini–Hochberg correction (Benjamini and Hochberg, 1995). GO categories with a false discovery rate (FDR) *P*-value < 0·05 were reported. The gene annotation data were taken from the header of the TAIR10 blast dataset.

### Statistical analysis

Kruskal–Wallis tests were performed using the statistical software R v. 2.13.1 (http://www.r-project.org/) to test for statistically significant differences between H and FS individuals from each population (KTP and WP) for the following traits: medial stamen length, lateral stamen length, pistil length, herkogamy for medial stamens, herkogamy for lateral stamens, ovule number, ovule area, total flowers per ramet, average pollen per flower, average seed number per silique, proportion of siliques with at least one seed, average seed number per ramet and clonal spread.

## RESULTS

### Variation in the frequency and morphology of the two morphs among populations

**Fig. 2.**
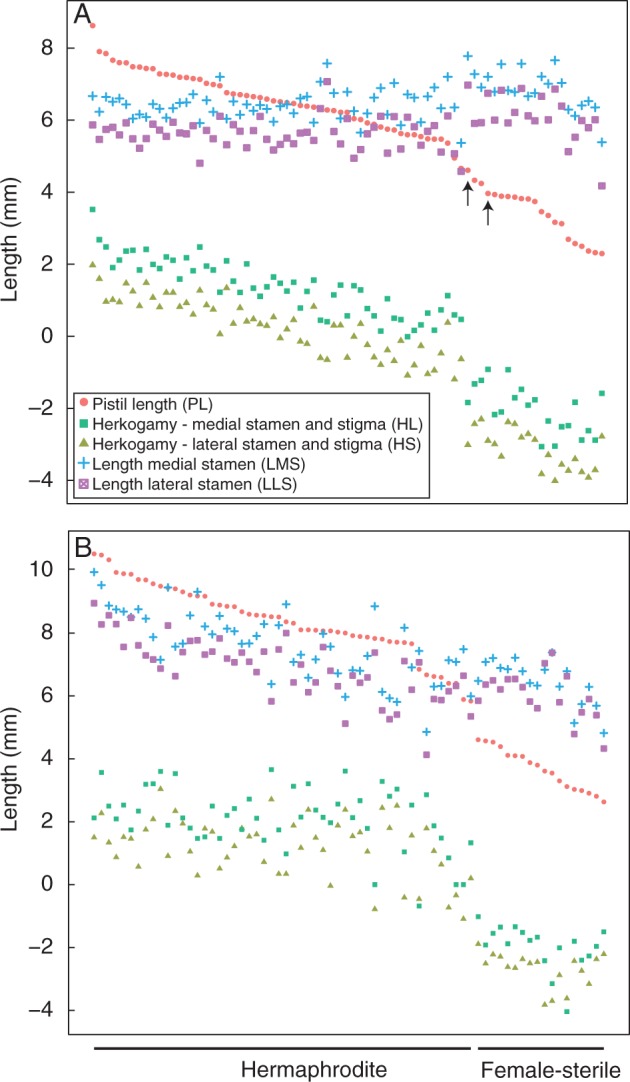
Distribution of floral morphological measurements at (A) KTP and (B) WP. Individuals are ranked according to pistil length (PL). Black arrows indicate FS individuals, which showed leaky female fertility at KTP.

The proportion of FS ramets varied greatly among populations, ranging from 0 % in the TP population to 54·4 % in the WP population (Table 1). Although pistil length varied continuously at both KTP and WP, there was nevertheless a fairly clear demarcation delimiting the heights of stigmas between H and FS ramets (Table 2 lines 1 and 2 and Fig. 2).

**Fig. 3.**
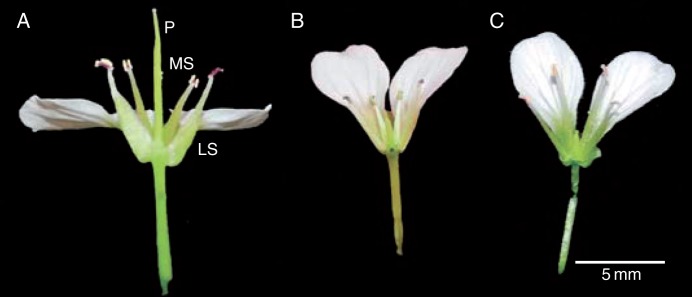
Variation in floral morphology in *Cardamine amara* flowers 2 d after anthesis. (A) Hermaphrodite from WP with typical floral ground plan. (B) Female-sterile flower from WP demonstrating reduced medial stamen length. (C) Female-sterile flower from KTP. P, pistil; MS, medial stamen; LS, lateral stamen. To aid visibility, two petals, two sepals and two medial stamen have been removed.

**Table 2.** Means (standard errors) of all parameters measured at KTP and WP for hermaphrodite and female-sterile individuals

Category		Trait		KTP			WP		
				Hermaphrodites	Female sterile	*P*	Hermaphrodites	Female sterile	*P*
Morphological measurements	1	Medial stamen length (LMS)		6·42 (0·39)	6·86 (0·57)	***	6·05 (0·51)	4·63 (1·46)	***
	2	Lateral stamen length (LLS)		5·62 (0·41)	6·09 (0·65)	***	5·4 (0·51)	4·79 (0·89)	***
	3	Pistil length (PL)		6·55 (0·82)	3·43 (0·71)	***	6·2 (0·78)	2·55 (0·84)	***
	4	Herkogamy of medial stamen (HL)		0·3 (0·79)	–3·15 (0·55)	***	0·29 (0·7)	–1·93 (0·9)	***
	5	Herkogamy of lateral stamen (HS)		1·38 (0·77)	–2·03 (0·66)	***	1·17 (0·58)	–1·96 (0·83)	***

Ovule measurements	6	Ovule number (average of five pistils)		27·63 (3·27)	22·33 (1·52)	***	27·21 (2·98)	21·42 (1·68)	***
	7	Ovule area (μm^2^)		2·01 × 10^4^ (234)	1·13 × 10^4^ (212)	***	2·01 × 10^4^ (273)	9·6 × 10^3^ (299)	***

Flower measurements	8	Total flowers per ramet		–	–	–	41·83 (12·1)	64·66 (29·07)	*
	9	Total flowers per genet^b^^,^^e^		43·5	193·98^b^	Not tested	61·49	230·84	Not tested

Pollen measurements	10	Average pollen per flower		–	–	–	1·77 × 10^4^	1·38 × 10^4^ (0·461 × 10^4^)	Not significant
							(0·696 × 10^4^)		
	11	Average pollen per ramet		–	–	–	7·04 × 10^5^	8·92 × 10^5^	Not tested
	12	Average pollen per genet^c^^,^^e^		7·32 × 10^5^^c^	2·68 × 10^6^^c^		1·03 × 10^6^	3·84 × 10^6^	Not tested
	13	Cross pollination as father (to hermaphrodite mother) Left, H × H; right, H × FS.	Average seed number per silique	2·18 (3·85)	1·22 (0·84	Not significant	3·73 (3·49)	1·4 (0·98)	*
	14		Proportion of siliques with at least one seed	0·44 (0·33)	0·21 (0·07)	Not significant	0·55 (0·39)	0·40 (0·28)	Not significant

Seed measurements	15	Cross pollination as mother Left, H × (H and FS); right, FS × (H and FS)	Average seed number per silique ^a^	1·8 (2·95)	0·17 (0·38)	***	2·57 (2·71)	0 (0)	***
	16		Proportion of silique with at least one seed	0·35 (0·27)	0·05 (0·12)	***	0·48 (0·33)	0 (0)	***
	17		Average seed number per ramet	–	–	–	44·5 (36·7)	0 (0)	***
	18		Average seed number per genet^d^^,^^e^	46·28^d^ (38·16)	0^d^ (0)	Not tested	65·42 (22·75)	0 (0)	Not tested
	19	Control (bagged, with no manual pollination)	Average seed number per silique	0·54 (0·67)	0 (0)	Not tested	0·16 (0·47)	0 (0)	Not tested
	20		Proportion of siliques with at least one seed	0·14 (0·16)	0 (0)	Not tested	0·06 (0·19)	0 (0)	Not tested
	21	Natural pollinations as mother	Average seed number when naturally pollinated	4·36 (3·18)	0 (0)	***	4·17 (2·9)	0 (0)	***
	22		Proportion of siliques with at least one seed when naturally pollinated	0·71 (0·27)	0 (0)	***	0·73 (0·19)	0 (0)	***
	23	Self pollinations Left, H × H (self); right, FS × FS (self)	Average seed number	1·16 (1·05)	0·22 (0·54)	***	2·99 (2·06)	0 (0)	***
	24		Proportion of siliques with at least one seed	0·38 (0·32)	0·01 (0·03)	***	0·55 (0·40)	0 (0)	***

Clonality	25	Clonal spread		1·04 (0·2)	3 (3·63)	**	1·47 (0·84)	3·57 (4·01)	Not significant
		Clonal diversity (*R*)		0·85	0·25	Not tested	0·35	0·11	Not tested
	26	Genet frequency		0·89	0·11	Not tested	0·71	0·29	Not tested
	27	Number of unique genotypes (number of tested ramets)		48 (53)	6 (18)	Not tested	15 (22)	6 (25)	Not tested

Genetic diversity	28	Estimated heterozygosity (*H*_E_)^f^		0·39			0·34		
	29	Observed heterozygosity (*H*_O_)^f^		0·48			0·51		
	30	Inbreeding coefficient (*F*_IS_)^f^		0·18 (−0·1 to 0·46)^g^			0·32 (0·06–0·58)^g^		
	31	Inferred selfing rate (*S*)^f^		0·31			0·49		

*P-*values were calculated using the Kruskal–Wallis test. For details of median, range, χ^2^ and actual *P*-values, see Supplementary Data Table S4.

–, measurement not taken at KTP

^a^Data from the field study

^b^Estimate of total flower number per genet at KTP based on total flower number per ramet measurements from WP.

^c^Estimate of average pollen per genet at KTP based on average pollen per ramet data collected at WP.

^d^Estimate of seed per genet at KTP based on seeds per ramet measurements from WP.

^e^Estimation assumed no difference between hermaphrodite and female-sterile individuals at WP and KTP.

^f^Estimates of genetic diversity were calculated at the population level, including both hermaphrodite and female-sterile genotypes.

^g^95 % confidence intervals are given in parentheses.

No inbreeding depression was assumed in inferring selfing rate.

*P < 0·05; **P < 0·01; ***P < 0·001.

To test whether pistil length variation between floral morphs could be explained by delayed development in FS pistils, we observed 20 flowering ramets (10 FS and 10 H) from WP at intervals for 4 days after flower opening. Within this period, the floral morphology of FS and H was relatively stable (Supplementary Data Fig. S1). Although pistil length increased over this period in both morphs, FS pistils were always much reduced compared with their H counterparts (Table 2 line 3) and did not show any delayed maturation.

Stamen length varied continuously within populations and tended to correlate positively with pistil length (Fig. 2). However, both median and lateral stamen lengths showed a discrete step in the otherwise continuous distribution among FS ramets within the KTP population, providing evidence that FS stamens were longer than those of the H ramets in the KTP population (Table 2 lines 1 and 2 and Fig. 2A). Furthermore, in the WP population lateral stamen length in a large proportion of FS ramets was similar to or actually greater than medial stamen length, in contrast to the typical tetradynamous condition of most Brassicaceae species (Figs 2B and 3).

### Female sterility in *C. amara*

At WP, both field observations of naturally pollinated ramets (Table 2 lines 21 and 22) and field pollination experiments (Table 2 lines 15 and 16) revealed that FS ramets did not produce any seed, whereas H ramets did. A similar pattern was observed at KTP, although two ‘leaky’ FS ramets produced a few seeds late in the flowering season (12 and 16 d after the first flower opened) (Table 2 lines 15, 16, 21 and 22). In H ramets, self- and cross-pollination resulted in similar numbers of seeds (Table 2 lines 15, 16, 23 and 24). Control bag treatments with no manual pollinations yielded only a small number of seeds (Table 2 lines 19 and 20), confirming that the bagging treatment minimized uncontrolled pollinations, and that autonomous self-pollination and/or apomixis had little effect. Phenotypic gender was consistent across ramets and genets over the entire inflorescence. It was also consistent across successive flowering seasons under controlled laboratory conditions.

### Differences between morphs in ovule number and morphology

**Fig. 4.**
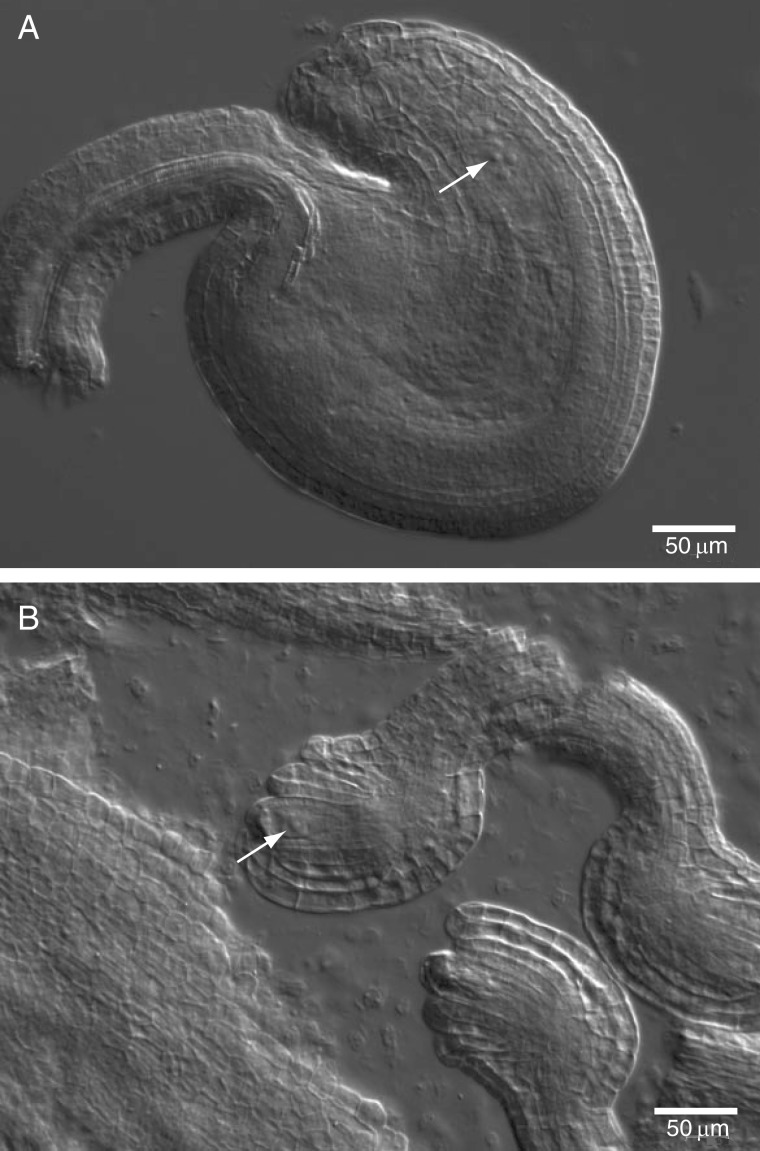
Ovules from 1-d-old flowers of *Cardamine amara*. (A) Ovules of a hermaphrodite flower have an embryo sac with an egg cell (arrow) and two unfused polar nuclei. (B) Ovules of female-sterile flower arrest in early development around the time of meiosis. The arrow indicates the megaspore produced immediately after meiosis.

Ovule number per pistil in FS and H were significantly different at both KTP and WP (Table 2 line 6). On average, FS ramets exhibited 19 % fewer ovules per pistil at KTP and 21 % fewer at WP compared with H ramets in the respective populations. Projected ovule area in FS was on average 46 % smaller than that of H at KTP (Table 2 line 7). To investigate the basis of ovule size reduction, we performed microscopic analysis of ovules from FS and H ramets for pistils of flowers on their first day of opening (Fig. 4). The results show that, for each of the ten FS ovules, development arrested at an early stage, likely when the megaspore mother cells underwent meiosis or very soon thereafter (Fig. 4B). In contrast, each of the ten H ovules tested had an embryo sac with a conspicuous nucleus of the egg cell (late FG5 stage) (Christensen *et al*., 1997; Shimizu *et al*., 2008), which could be fertilized (Fig. 4A).

### Male fertility in female-sterile and hermaphrodite individuals

Field observations at WP revealed that FS ramets produced an average of 54 % more flowers than H ramets (*P* < 0·05) (Table 2 line 8). Pollen number per flower (averaged over five flowers per individual) was 28·2 % higher in H ramets than FS ramets, although the difference was not significant (Table 2 line 10). When combined, FS ramets produced 17 % more pollen per ramet than H ramets (Table 2 line 11). 

**Fig. 5.**
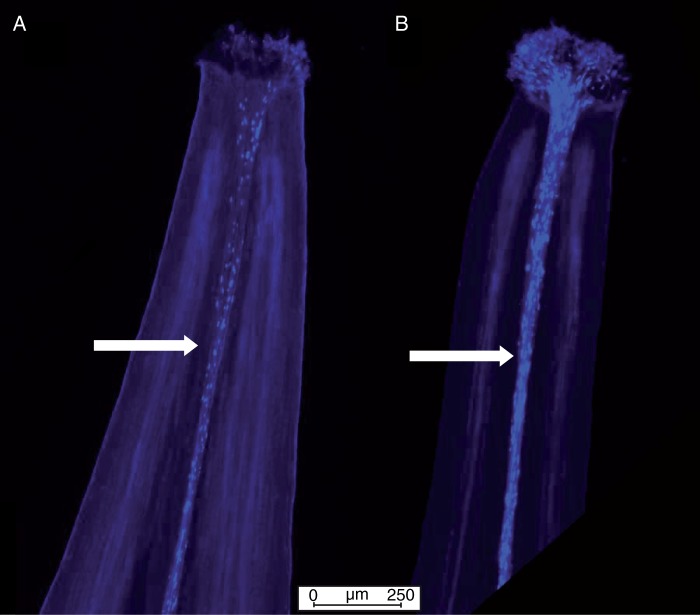
Epifluorescence micrographs showing pollen viability of female-sterile pollen on a hermaphrodite stigma (A) and hermaphrodite pollen on a female-sterile stigma (B). Pollen tubes are clearly visible in both images in fluorescent blue. Pollen tubes are indicated by arrows.

Epifluorescence microscopy revealed that pollen from both FS and H ramets was capable of producing functional pollen tubes (Fig. 5). In the manual pollination experiments in the field, pollen from both H and FS ramets generated seeds, confirming the fertility of FS pollen (Table 2 lines 13 and 14). We found no significant differences in seed production when FS and H pollen was used at KTP. We observed a tendency towards lower seed production following pollination by pollen from FS ramets compared with H ramets, particularly when average seed number per silique at WP was compared, although the variance for both WP and KTP was high and statistical support was not strong (Table 2 lines 13 and 14).

### Mating system and clonal propagation

In the field crossing experiments, we found no apparent barrier to self-fertilization in hermaphrodites at either WP or KTP, and seed production from self and outcross pollination was approximately equal, suggesting self-compatibility in the two populations. Microsatellite data from eight polymorphic loci (Supplementary Data Table S1) pointed to intermediate selfing rates in wild populations (i.e. mixed mating) (Table 2 lines 30 and 31).

Although we employed a sampling strategy that attempted to avoid sampling multiple ramets from any single genet, some multilocus genotypes were shared within populations. In the WP population, 15 unique multilocus genotypes were observed in H individuals (Table 2 line 27) and only six unique multilocus genotypes were observed in FS genotypes. Of these six genotypes, two were sampled across the population nine and ten times, respectively, from a total of 47 sampled ramets. The probability that these FS ramets had the same genotype as a result of sexual production was *p*_sex_ = 8·79 × 10^–^^17^ and 2·68 × 10^–^^30^, respectively. Analogously, in the KTP population we found 48 unique multilocus genotypes in H individuals and only six in FS individuals (Table 2 line 27), two of which were sampled four and ten times, respectively, from a total of 71 sampled ramets, with *p*_sex _= 2·2 × 10^–^^79^ and 1·8 × 10^–^^78^, respectively. Clonal diversity estimates (*R*) between morphs revealed that FS individuals in both populations had lower clonal diversity than H individuals in both populations (Table 2 line 25). When the frequency of ramets with identical genotypes was used to calculate mean clonality in both morphs, we estimate that FS individuals produced 2·9 and 2·4 times more ramets per genet than H individuals in the KTP and WP populations, respectively (Table 2 line 25). Using these data, we calculated that per genet female-sterile/FS frequency was 11 % at KTP and 29 % at WP (Table 2 line 26).

Individuals with shared genotypes always had the same phenotypic gender in both populations, pointing to the developmental stability of the sterility trait. Additionally, when seeds from a single H mother from WP were grown in the greenhouse, we observed both morphs in the progeny (three H individuals and two FS individuals) in spite of a low germination rate. When seeds from a single H mother from KTP were grown, similarly, two H and three FS individuals were observed. These data suggest that this trait is not maternally inherited but is likely to be autosomal, and there is little doubt that the two morphs are members of the same reproductive unit.

### Differences in gene expression between morphs

**Fig. 6.**
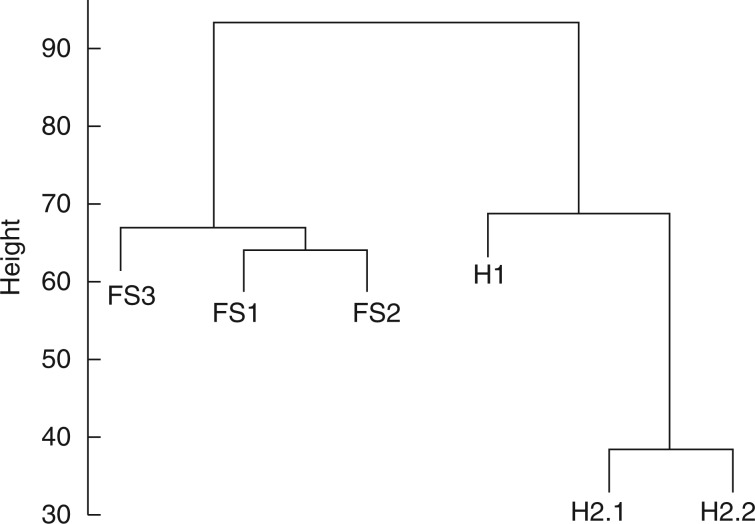
Hierarchical agglomerative cluster analysis of the expression pattern of 11 900 genes after removing all genes that did not show 100 % binding to the microarray chip. H1, H2.1 and H2.2 represent hermaphrodites, and FS1, FS2 and FS3 represent female-sterile individuals. The three biological replicates of each morph cluster together.

Because microscopic analysis revealed arrested ovule development, we hypothesized that the gene expression of many genes may be different in H and FS pistils. Indeed, cluster analysis showed that expression data from the three H and three FS replicates formed separate clusters (Fig. 6). Moreover, 76 genes showed differential expression between the two morphs above a significance threshold (log_2_ fold change of ≥3) (Supplementary Data Table S2). The list includes well-studied genes with diverse functions, such as *DEHYDRATION-RESPONSIVE ELEMENT BINDING PROTEIN 2B* (*DREB2B*) in dehydration and salinity response (Nakashima *et al*., 2000), *INDOLE-3-ACETIC ACID INDUCIBLE 12/BODENLOSS* (*IAA12/BDL*) in auxin response (Hamann *et al*., 2002), *P-GLYCOPROTEIN 4/ATP-BINDING CASSETTE B4* (*PGP4/ABCB4*), encoding auxin transporter (Terasaka *et al.*, 2005) and *CYTOCHROME P450, FAMILY 83, SUBFAMILY A, POLYPEPTIDE 1* (*CYP83A1*), involved in both glucosinolate biosynthesis and auxin homeostasis (Bak and Feyereisen, 2001) (Supplementary Data Table S2). Possibly reflecting its diversity, only two GO terms (glucosinolate biosynthetic process and hyperosmotic salinity response) were enriched in the gene list (Supplementary Data Table S2).

## DISCUSSION

### Female sterility in *C. amara* and its molecular and developmental basis

Our study has found that *C. amara* populations in Switzerland show dimorphism in the propensity for seed production and in associated morphological and life-history traits. Thus, populations comprise (1) self-compatible H individuals (or genets) with typical floral morphology and development, and (2), at lower frequency, FS individuals that show aberrant carpel and ovule development and shorter pistils, and a greater propensity to vegetative propagation via clonal spread. The floral ground plan of some FS individuals at KTP varies from that of typical Brassicaceae species [tetradynamous condition – four long ‘medial’ and two short ‘lateral’ anthers in each flower (Endress, 1992; Zomlefer, 1994)], although variation in male morphological positioning of sex organs is nevertheless largely continuous. Because both FS and H progeny segregated from a single H mother following controlled crossing, purely cytoplasmic inheritance of female sterility can be ruled out, as can the possibility that the two morphs represent reproductively isolated taxa (though further verification with larger samples would be valuable). The variation observed would thus appear to be a clear genetic polymorphism, likely segregating at one or more autosomal loci, although again this suggestion could benefit from wider testing.

The differences we have identified between the H and FS morphs were consistent and effectively diagnostic of a dimorphic gender strategy. Importantly, the gender expression of specific ramets and genets was maintained over all flowers of their inflorescences as well as across successive flowering seasons. It would thus seem unlikely that environmental factors (such as temperature) play a decisive role in the differential development of H versus FS flowers, in contrast with the environmental influences on floral development observed in *Cardamine hirsuta* (Matsuhashi *et al*., 2012). Nevertheless we did find two FS individuals that produced a few seeds. These likely represent ‘leaky females’, a phenomenon observed in many dioecious and subdioecious species (Lloyd, 1976; Dorken and Barrett, 2004; Ehlers and Bataillon, 2007; Barrett, 2010), and do not reflect the existence of a continuum in gender expression between H and FS individuals, as found, for example, in several plant species with a tendency towards separate sexes [including *Sagittaria latifolia* (Dorken and Barrett, 2004); *Wurmbea dioica* (Barrett, 1992) and *Cotula dioica* (Lloyd, 1975*b*)]. So-called leakiness in gender expression is in fact a common phenomenon in species with fully dimorphic gender strategies, with males of dioecious species that have evolved via gynodioecy often producing a few female flowers (Delph and Wolf, 2005). Our discovery of female sterility segregating in populations of *C. amara* is, to our knowledge, the first report of such a phenomenon in the Brassicaceae.

We observed flower development at intervals between flower opening and senescence, and found that pistils of FS individuals were already much shorter than those of H individuals at the time of flower opening (Supplementary Data Fig. S1). We used arabidopsis microarrays (Morinaga *et al*., 2008) to compare genome-wide expression patterns between the two morphs of *C. amara* at the stage when the style length difference became discernable in the bud (Supplementary Data Fig. S1) and revealed separate expression clusters for the two morphs (Fig. 6). This further supports the distinctiveness of the two morphs in terms of their patterns of gene expression. A list of genes regulated differentially between the two morphs (Supplementary Data Table S2) provides putative candidate genes associated with female sterility, although our simple analysis of gene ontology has not revealed any significant enrichment of genes for developmental regulation or cell death (Supplementary Data Table S3). Female sterility could ultimately be caused by any defect in pistil development, physiology or metabolism. Given that the plant hormone auxin is an important regulator of pistil development (reviewed by Ståldal and Sundberg, 2009), it is perhaps interesting that we did observe differential expression of genes involved in auxin response or transport.

### Trade-offs between reproduction and clonal propagation in *C. amara*

We found that FS individuals showed a 2·4- to 2·9-fold greater rate of clonal spread (i.e. production of new ramets) than H individuals and produced 57 % more flowers per ramet. These differences are probably conservative estimates because we sampled only a single individual per patch at intervals of >2 m to reduce the likelihood of sampling clones of the same individual. The increased performance in floral and vegetative productivity is a likely outcome of trade-off through diversion of resources away from fruit production in FS individuals. Trade-offs between reproduction and growth are well documented in plants in general (e.g. Harper, 1977; Wilson and Burley, 1983; Ågren and Wilson, 1994; Silvertown and Dodd, 1999; Obeso, 2002) and between reproduction and clonal propagation in particular (Dorken and Eckert, 2001; Thompson and Eckert, 2004).

The increased clonal size and floral display of FS genets seem likely to confer fitness benefits through their interactions with pollinators and associated siring opportunities First, the greater floral displays of FS genets with multiple ramets might enhance their ability to attract pollinators and allow them to package and/or disperse their pollen more effectively (Lloyd and Yates, 1982; Bell, 1985; Harder and Thomson, 1989; Young and Stanton, 1990; Wilson *et al*., 1994; Castellanos *et al*., 2006; Glaettli and Barrett, 2008). The opportunity to successfully sire offspring is limited to the time when receptive flowers are available to pollen, thus presenting a temporal component to mating success. By spreading their flowers over multiple ramets at potentially different times, FS individuals could spread their exposure to pollinators over space and maximize mating opportunities by presenting pollen gradually (Harder and Thomson, 1989; Wilson *et al*., 1994).

In general, spreading the presentation of pollen over space and time will tend to linearize the male fitness gain curve (Wilson *et al*., 1994), easing the selective constraints on the evolution of male phenotypes (Dorken and van Drunen, 2010). The fragmentation of ramets of a large genet in *C. amara* could potentially enhance male siring success of FS genets if each ramet is able to take advantage of the steepest parts of the male fitness gain curve, particularly if the fitness gain curve is strongly saturating (Dorken and van Drunen, 2010). A similar argument was advanced to explain the sexual system of the aquatic plant *S. latifolia*, in which males produce around twice as many flowers as hermaphrodites, but they display only a few open flowers at any given time, increasing their mating opportunities and reducing the mating cost of large floral displays (Perry and Dorken, 2011).

### The combination of androdioecy and clonal propagation in *C. amara*

Competition for reproductive resources in perennial plants can give rise to significant variation in the balance between sexual and asexual (clonal) reproduction. For instance, in the aquatic species *Decodon verticillatus* (Lythraceae)*,* reduced fertility of both the male and female components appears to be associated with a switch to clonal propagation in certain populations (Dorken and Eckert, 2001). To what extent is the system of *C. amara* sexual versus purely asexual?

It is clear from our results that both FS and H individuals of *C. amara* have at least partially functional male components. In both populations studied, open pollination in the field resulted in the production of a mean of approximately four seeds per fruit on H individuals (Table 2 line 21). It is therefore clear that natural pollination results in the transfer of viable pollen onto H stigmas. Measurement of seed set resulting from hand pollination in the field confirmed the viability of *C. amara* pollen; indeed, pollen from both FS and H individuals was able to sire seeds, although seed set was generally lower than under natural pollination of H individuals.

Cellular observations using epifluorescence microscopy after hand pollination in the laboratory also confirmed the viability of pollen, with pollen from both morphs germinated and pollen tubes growing apparently normally. Finally, the fact that the populations sampled contained a number of multilocus microsatellite genotypes provides a clear indication that reproduction in *C. amara* is not purely asexual and involves successful siring by pollen. Continued sexual reproduction in plant populations with substantial asexual ramet proliferation is very common in perennial plants (Dorken and Eckert, 2001; Silvertown, 2008). However, the reproductive behaviour of *C. amara* is particularly interesting, because asexuality appears to be associated with reduced sexual fertility, as has been found in studies of the aquatic perennial *D. verticillatus* cited above (Dorken and Eckert, 2001).

The extreme rarity in flowering plants of female sterility / FS in otherwise hermaphroditic populations (androdioecy) has been attributed to the difficulty that males have in siring progeny among individuals that produce both pollen and seeds, particularly if these individuals self-fertilize a proportion of their progeny (Lloyd, 1975*a*; Charlesworth and Charlesworth, 1978; Charlesworth, 1981, 1984). For FS individuals to invade and spread among hermaphrodites, they must sire at least twice as many successful progeny as do the hermaphrodites. To some extent, the expression of inbreeding depression by selfed individuals can ease the conditions for their maintenance, but selfing by hermaphrodites ultimately always makes coexistence more difficult (Ishida and Hiura, 2002; Pannell, 2002; Eppley and Pannell, 2007). Our results indicate that selfing is reasonably common in *C. amara*, so the maintenance of FS individuals in its populations would seem to be particularly surprising. In androdioecious *Datisca glomerata*, in which males co-occur with hermaphrodites, pollen production per flower by males greatly exceeds that by hermaphrodites (Liston *et al.*, 1990; Philbrick and Rieseberg, 1994; Spencer and Rieserberg, 1995), and in *Mercurialis annua* (Pannell, 1997) and *Schizopepon bryoniifolius* (Akimoto *et al*., 1999) males produce many times more flowers than hermaphrodites. However, these observations contrast with those for *C. amara*, in which pollen production per flower by FS ramets appears to be similar to or lower than that of H individuals, and the number of flowers per ramet appears to be only ∼50 % greater. How, then, might female sterility be maintained?

Although sexual reproduction is being maintained in *C. amara*, there seems little doubt that the greater clonal reproduction of FS individuals of *C. amara* plays a large part in maintaining the morph in populations by compensating for its loss in fitness through seed production. Critically, theoretical predictions for the maintenance of female sterility assume that all reproduction is sexual and do not account for the advantages that genotypes might gain through asexual proliferation. In such cases, increased proliferation of female sterility, as observed for the FS morph in *C. amara*, is likely to contribute to its maintenance. Consistent with the theoretical prediction of siring success described above, the average amount of pollen per genet of FS individuals is estimated to be more than twice that of H individuals (Table 2 line 12) due to higher clonal propagation, higher pollen number per flower and higher flower number per ramet.

### Conclusions and final remarks

To summarize, the following data on the reproductive system of these unusual populations of *C. amara* point to an unusual combination of androdioecy and asexuality in some of its populations; the case may be similar in some respects to the maintenance of FS individuals in clonal populations of *Ulmus minor* (López-Almansa *et al*., 2002). (1) Female-sterile individuals exhibit arrested ovules in short pistils, and show altered transcriptomes, consistent with their female sterility. Crosses in the field also confirmed the female sterility, while rare leaky FS individuals were also observed. (2) Seed set resulting from hand pollination in the field confirmed the functionality of the male components of FS individuals as well as the male fertility of hermaphrodites, indicating that the reproductive system of *C. amara* is not a case of cryptic dioecy. (3) Manual pollination in the laboratory showed viability of pollen tubes for both FS and H individuals, supporting the above data from the field. (4) The diversity of the multilocus microsatellite genotypes exclude the possibility that *C. amara* is purely asexual. (5) Clones with identical multilocus genotypes always had the same gender, supporting the genetic determination of gender, rather than phenotypic plasticity. (6) Under controlled laboratory conditions, phenotypic gender (female sterility versus hermaphroditism) was constant across successive flowering seasons, which also supports the genetic determination of the gender. (7) Female-sterile and hermaphrodite individuals were derived from single mothers and thus belong to the same reproductive unit, although sampling on a larger scale would corroborate this inference. (8) Higher clonal propagation by FS individuals compared with hermaphrodites was confirmed by microsatellite genotyping. The genet frequency of FS individuals (11–29 %) was <50 %, which is the theoretical maximum predicted by simple theories of androdioecy. (9) Most importantly, due to a high clonal propagation rate, FS genets produced more than double the amount of pollen grains compared with hermaphrodites, which is a necessary condition under simple models for the maintenance of FS genets predicted by such androdioecy theories.

Androdioecy has tended to be considered in the context of gender polymorphism maintained as the outcome of male versus hermaphrodite siring success, so canonical models are not entirely adequate for accounting for its maintenance in *C. amara*. New models would be useful. From an empirical point of view, we still have little data on the relative contributions of sexual and asexual reproduction of FS individuals in *C. amara*. It is also not known how widespread female sterility in the species is, how population structure affects patterns of mating and eventual inbreeding depression, or how the sterility is determined genetically. Further empirical work will be extremely valuable in addressing these outstanding questions. 

Because of its close relationship to the genomic model plant *A. thaliana*, *C. amara* presents a unique and exciting opportunity for studying androdioecy and clonal reproduction, not only because sexual and asexual reproduction persist together, but especially because of the differences we have observed between the two morphs in terms of their likely contributions through the two modes of reproduction. *Cardamine amara* also presents a potentially interesting model for the study of the advantages and disadvantages of sexual and asexual reproduction. The mix of sexual with clonal reproduction might represent a fortuitous state for the long-term maintenance of the asexual strategy, given that small amounts of sex and recombination are likely to be sufficient to counteract some of the disadvantages of asexuality (Otto and Lenormand, 2002; Pannell, 2009; D’Souza and Michiels, 2010).

## SUPPLEMENTARY DATA

Supplementary data are available online at www.aob.oxfordjournals.org and consist of the following. Figure S1: floral traits measured on individuals from WP over 4 successive days after flower opening. Table S1: microsatellite primer range and diversity (number of alleles) in *Cardamine amara*. Table S2: list of top candidate genes in immature pistils that are expressed differentially between hermaphrodite and female-sterile individuals. Table S3: GO terms reflecting biological processes that are significantly overrepresented. Table S4: medians, ranges and Kruskal–Wallis χ^2^ values of all parameters measured at KTP and WP for hermaphrodites and female-sterile individuals.
